# Using oral fluids samples for indirect influenza A virus surveillance in farmed UK pigs

**DOI:** 10.1002/vms3.51

**Published:** 2016-12-16

**Authors:** Priscilla F. Gerber, Lorna Dawson, Ben Strugnell, Robert Burgess, Helen Brown, Tanja Opriessnig

**Affiliations:** ^1^ The Roslin Institute and The Royal (Dick) School of Veterinary Studies University of Edinburgh Midlothian Scotland UK; ^2^ School of Agriculture Food and Rural Development Newcastle University Newcastle upon Tyne UK; ^3^ Evidence‐based Veterinary Consultancy (EBVC) Ltd. Rural Enterprise Centre Redhills Penrith Cumbria UK; ^4^ Department of Veterinary Diagnostic and Production Animal Medicine Iowa State University Ames Iowa USA

**Keywords:** Swine influenza virus, diagnostics, enzyme‐linked immunosorbent assay, surveillance

## Abstract

Influenza A virus (IAV) is economically important in pig production and has broad public health implications. In Europe, active IAV surveillance includes demonstration of antigen in nasal swabs and/or demonstration of antibodies in serum (SER) samples; however, collecting appropriate numbers of individual pig samples can be costly and labour‐intensive. The objective of this study was to compare the probability of detecting IAV antibody positive populations using SER versus oral fluid (OF) samples. Paired pen samples, one OF and 5–14 SER samples, were collected cross‐sectional or longitudinally. A commercial nucleoprotein (NP)‐based blocking ELISA was used to test 244 OF and 1004 SER samples from 123 pens each containing 20–540 pigs located in 27 UK herds. Overall, the IAV antibody detection rate was higher in SER samples compared to OFs under the study conditions. Pig age had a significant effect on the probability of detecting positive pens. For 3–9‐week‐old pigs the probability of detecting IAV antibody positive samples in a pen with 95% confidence intervals was 40% (23–60) for OF and 61% (0.37–0.80) for SER (*P *=* *0.04), for 10–14‐week‐old pigs it was 19% (8–40) for OF and 93% (0.71–0.99) for SER (*P *<* *0.01), and for 18–20‐week‐old pigs it was 67% (41–85) for OF and 81% (0.63–0.91) for SER (*P *=* *0.05). Collecting more than one OF sample in pens with more than 25 less than 18‐week‐old pigs should be further investigated in the future to elucidate the suitability of OF for IAV surveillance in herds with large pen sizes.

## Introduction

Influenza A virus (IAV) in contemporary commercial swine populations is an important endemic disease with significant cost at the herd level along with broad public health implications (Detmer *et al*. 2013). In domestic pigs, IAV is one of the main respiratory pathogens with a resultant weight loss or reduced weight gain in growing pigs and, in some cases, reproductive failure in infected sows due to systemic illness and high fevers (Rajao *et al*. 2014). Pigs are susceptible to both avian and human IAVs and the swine IAVs can be transmitted from pigs to other species, including humans (Kuntz‐Simon & Madec 2009). Because of public health concerns, some geographical areas initiated IAV monitoring in swine. Surveillance for IAV can be passive, i.e. submission of samples is initiated when respiratory disease is observed, or active, i.e. based on purposeful collecting and screening of field samples regardless of clinical status (Simon *et al*. 2014). Since 1991, the Animal and Plant Health Agency (APHA) has conducted passive surveillance for swine IAV in the UK by reverse‐transcriptase (RT) PCR testing of samples from pigs with respiratory disease (Williamson *et al*. 2012).

Whether the focus is swine production or public health, the challenge with IAV monitoring in swine populations is collecting a sufficient number of samples at the right time during the course of the infection (Detmer *et al*. 2013; Vincent *et al*. 2014). Testing of pig nasal swabs by virus isolation or real‐time reverse transcriptase (rRT)‐PCR is commonly done to demonstrate an acute IAV infection. In contrast, serum (SER) samples are regularly used to demonstrate antibodies against IAV by haemagglutination inhibition (HI) assay or ELISA (Detmer *et al*. 2013). Not all pigs may present respiratory disease typical of IAV infection making acquisition of appropriate samples for use in rRT‐PCR assays difficult (Grontvedt *et al*. 2011; Buehler *et al*. 2014). Serological assays provide a number of benefits compared to molecular detection of IAV, the most important one being the ability to detect IAV exposure after active viral replication has ceased (Buehler *et al*. 2014; Panyasing *et al*. 2016). While IAV RNA can be demonstrated in lung tissues and nasal swabs for approximately 7 days (Heinen *et al*. 2001; Rose *et al*. 2013), antibody detection in SER samples starts approximately 7–10 days after initial exposure and antibodies can persist for at least 6–8 weeks (Heinen *et al*. 2001; Barbe *et al*. 2009; Rose *et al*. 2013). In Europe, demonstration of IAV antibody in SER is preferred for active IAV surveillance (von Dobschuetz *et al*. 2015) and presence of IAV specific antibody is indicative of previous IAV exposure in pigs with no vaccination history (Fragaszy *et al*. 2015). It is recommended to follow up IAV antibody positive pigs from suspect outbreaks by RT‐PCR to confirm the presence of IAV RNA (von Dobschuetz *et al*. 2015). In programmes aiming to eliminate IAV in a given herd, serology can be used to rule out an ongoing active IAV infection (Detmer *et al*. 2013). Serological screening of asymptomatic herds can also aid to detect uncommon IAV strains (Simon *et al*. 2014) as the chances of detection of positive animals are higher when compared with RT‐PCR testing. In addition, IAV serology is commonly used to evaluate vaccine immunogenicity (Hughes *et al*. 2015). Sero‐surveillance studies conducted in England based on testing of 2745 weaned, grow‐finish and sow SER samples from 143 farms found that 52% of the farms and 12% of the animals were positive for antibodies against at least one strain of IAV (Mastin *et al*. 2011).

The rapid evolution of IAV has led to genetic and antigenic variation in the virus (Simon *et al*. 2014; Anderson *et al*. 2015). Due to limited cross‐reaction between swine IAV subtypes, multiple viruses from each subtype are needed for HI assays, which complicates routine diagnostics and surveillance (Detmer *et al*. 2013; Goodell *et al*. 2016) and adds costs. To overcome the limitations of cross‐reactivity and to broaden IAV surveillance, a nucleoprotein (NP)‐blocking ELISA originally designed for use in avian species has been implemented successfully in pig diagnostics (Ciacci‐Zanella *et al*. 2010; Goodell *et al*. 2016; Panyasing *et al*. 2016). The sensitivity and specificity of the test on SER samples was estimated to be 96.6% and 99.3%, respectively (Ciacci‐Zanella *et al*. 2010). The advantages offered by the ELISA format over the HI platform include a rapid throughput, higher repeatability and better quality control. Limitations of the NP‐blocking ELISA include the inability to differentiate between antibodies elicited by vaccines that contain IAV NP and field IAV infection and the inability to differentiate infections induced by different IAV subtypes. Nonetheless, there is a general trend towards use of commercially available ELISAs that are not subtype specific (OIE, 2015) for screening purposes, followed by more specific tests, such as HI assays, to determine the subtype of the involved IAV strain.

While individual pig sampling is adequate for the diagnosis of clinical IAV infections, the collection of appropriate numbers of individual pig samples is too costly and labour‐intensive for IAV surveillance or large epidemiological studies. A study indicated that oral fluid (OF) sampling could be a useful tool for IAV surveillance in swine populations by allowing testing of large sample numbers in a cost‐effective manner (Panyasing *et al*. 2016). However, in the latter study a comparison between pen‐based OF and SER samples was not conducted. The objective of this study was to compare the prevalence of anti‐IAV antibodies in paired OF and SER samples by an IAV ELISA in 3–20‐week‐old UK pigs and with various levels of exposure to IAV.

## Material and methods

### Farms, experimental design and samples collected

Twenty‐seven commercial pig herds located in the UK were included in this study. All samples utilized originated from routine herd health monitoring for porcine reproductive and respiratory syndrome virus (PRRSv) (Dawson 2015). The protocol for this study was approved by the Newcastle University Institutional Biosafety Committee. Paired SER and OF samples were collected from all herds; 70.4% (19/27) herds were sampled on one occasion only and the remaining 29.6% (8/27) of the herds were visited at the time of weaning until around 12 weeks of age in approximately 2 week intervals. While OF samples were collected at each time point, SER samples were collected during the last visit only. All sample collections were performed between January and December 2013. The number of pens sampled within a herd, the number of pigs bled within a pen and the housing system are summarized in Table 1. Herds contained pigs of different ages (Table 1). Specifically, 7/27 farms housed 3–9‐week‐old pigs, 7/27 farms housed 18–20‐week‐old pigs and 13/27 farms contained 3–20‐week‐old pigs. The number of serum samples collected per pen (*n *=* *5–14 pigs) was calculated based on the ability to detect at least one seropositive animal at an assumed pen seroprevalence of 20–40% and with a confidence of 95% (Dawson 2015). A total of 1004 SER samples and 244 OF samples using cotton ropes were collected and processed as previously described (Dawson 2015). For the cross‐sectional study, OF samples were collected at a single point totalling 86 pens sampled. For the longitudinal study, 158 OF samples were collected from 37 pens. Samples were aliquoted into 1.5 mL microcentrifuge tubes and stored at −80°C until processing.

**Table 1:** Farms and number of serum (SER) samples collected per pen. One oral fluid sample was collected from each pen

Farm	N. pens sampled/Total n. pens	N. SER samples per pen/Average n. pigs per pen (%)	Age (weeks)	Housing
Longitudinal study				
L1	7/8	14/250 (6)	4	Straw
L2	3/6	13/190 (7)	4	Straw
L3	2/40	14/450 (3)	6	Straw/Slatted
L4	6/8	14/240 (6)	4	Straw
L5	5/20	14/390 (4)	5	Straw
L6	6/6	11/270 (4)	3	Straw
L7	5/9	14/232 (6)	5	Straw
L8	4/20	14/280 (5)	3	Straw
Cross‐sectional study				
C1	3/6	7/175 (4)	8	Straw
C3	10/20	5/50 (10)	8 to 18	Slatted
C4	3/8	8/300 (3)	8	Straw
C5	8/10	6/50 (12)	18	Straw
C8	4/20	3/30 (10)	18	Slatted
C9	4/10	6/300 (2)	8	Straw
C10	6/10	6/175 (3)	18	Straw
C11	6/10	6/200 (3)	8	Straw
C12	8/4	6/250 (2)	18	Straw
C14	3/20	6/125 (5)	8	Straw
C15	7/30	5/200 (3)	18	Straw
C16	2/40	6/250 (2)	8	Straw
C17	3/20	5/160 (3)	18	Straw
C18	5/40	6/25 (24)	12 to 18	Slatted
C19	2/40	7/25 (28)	18	Slatted
C23	5/40	6/20 (30)	8 to 18	Slatted
C24	3/15	7/100 (7)	12 to 20	Outdoor
C25	3/40	5/20 (25)	6 to 18	Slatted
C32	4/20	7/100 (7)	8	Straw

### NP‐blocking ELISA

Serum and OF samples were tested for NP antibodies using a commercial blocking ELISA (IDEXX Influenza A Ab Test; IDEXX Laboratories, Inc.). SER samples were tested for the presence of IAV antibodies as recommended by the manufacturer. The same commercial blocking ELISA kit was used to detect anti‐IAV antibodies in OF samples but the protocol was modified as previously described (Panyasing *et al*. 2014). Briefly, the main modifications included that each plate was loaded with 200 *μ*L undiluted OF and incubated for 16 h at 21°C. Serum and OF reactions were measured as optical density (OD) at a wavelength of 650 nm using an ELISA plate reader. Sample‐to‐negative (S/N) ratios were calculated as described by the manufacturer, with S/N ratios of ≤0.60 considered antibody positive. For the OF test interpretation, in addition to the S/N ratio cut‐off of ≤0.60 as suggested for SER samples by the manufacturer, results were also obtained and evaluated using a S/N ratio cut‐off of ≤0.65 as suggested by a previous study (Panyasing *et al*. 2014).

### RNA extraction and real‐time RT‐PCR

At least two OF samples were selected per farm for detection of IAV RNA by rRT‐PCR (*n *=* *92). For farms included in the longitudinal study, a sample that showed a rise in IAV antibody level and the sample collected just before this collection in the same pen were chosen for rRT‐PCR testing. In pens in which no rise in IAV antibody levels was noticed, the last collection point was selected for rRT‐PCR testing. Total nucleic acids were extracted using the MagMAX™ Viral RNA Isolation Kit (Life Technologies) according to the manufacturer's recommendations. The rRT‐PCR assay based on the TaqMan Fast Virus 1‐Step Master Mix (Life Technologies) to detect the IAV matrix gene using primers (IAV M + 25: 5′‐ AGATGAGTCTTCTAACCGAGGTCG ‐3′; IAV M‐124: 5′‐ TGCAAAAACATCTTC AAGTCTCTG ‐3′) and a probe (IAV M + 64: 5′‐6‐FAM‐ TCAGGCCCCCTCAAAGCCGA‐3′ BHQ‐1) was done as described previously (Spackman & Suarez 2008). Amplification reactions were performed using an Applied Biosystems 7500 Fast Real‐Time PCR System (Applied Biosystems) under universal conditions: 5 min at 50°C, 20 s at 95°C, followed by 40 cycles of 3 s at 95°C and 30 s at 60°C. A sample was considered positive when a threshold cycle (Ct) of < 38 was obtained.

### Statistical analysis

Analyses were carried out using the GLIMMIX procedure within the SAS software (SAS^®^ Version 9.4, SAS^®^ Institute, Inc., Cary, NC). Statistical modelling was used to estimate the probability of a positive test result by both methods and to assess the influence of factors such as animal age, pen and animal sampling rates, pen and herd sizes and housing type in IAV positive herds. Positive herds were defined as those for which there were two or more positive test results on pen‐based OF samples (S/N ratio cut‐off of ≤0.65) or two or more positive test results for individual SER samples. For confirmed positive herds, a positive pen test was defined as an IAV antibody positive OF sample with an S/N ratio ≤0.65 or with at least one positive SER sample. Only pens that were tested by both methods were included in the analyses. Generalised linear mixed models (GLMMs) were used for all analyses fitting herd as a random effect to allow for correlated results within herds. Factors such as animal age, pen sampling proportion, animal sampling rate, number of samples collected per pen, pen size, herd size, housing type, and their interactions with test type (i.e. whether the factor had an effect depending on test type) were considered fixed effects. Models were fitted (1) to estimate the probability of a positive pen result based on testing OF or SER samples using all results from the longitudinal studies for OF only along with the cross‐sectional studies; (2) to estimate the probability of a positive SER result for an individual pig which allowed to estimate the probability of a pen being considered positive when varying numbers of animals were tested; and (3) to estimate the probability of a positive OF result at any time point during longitudinal studies compared to SER samples.

## Results

### IAV status in longitudinal herds

**Figure 1:**
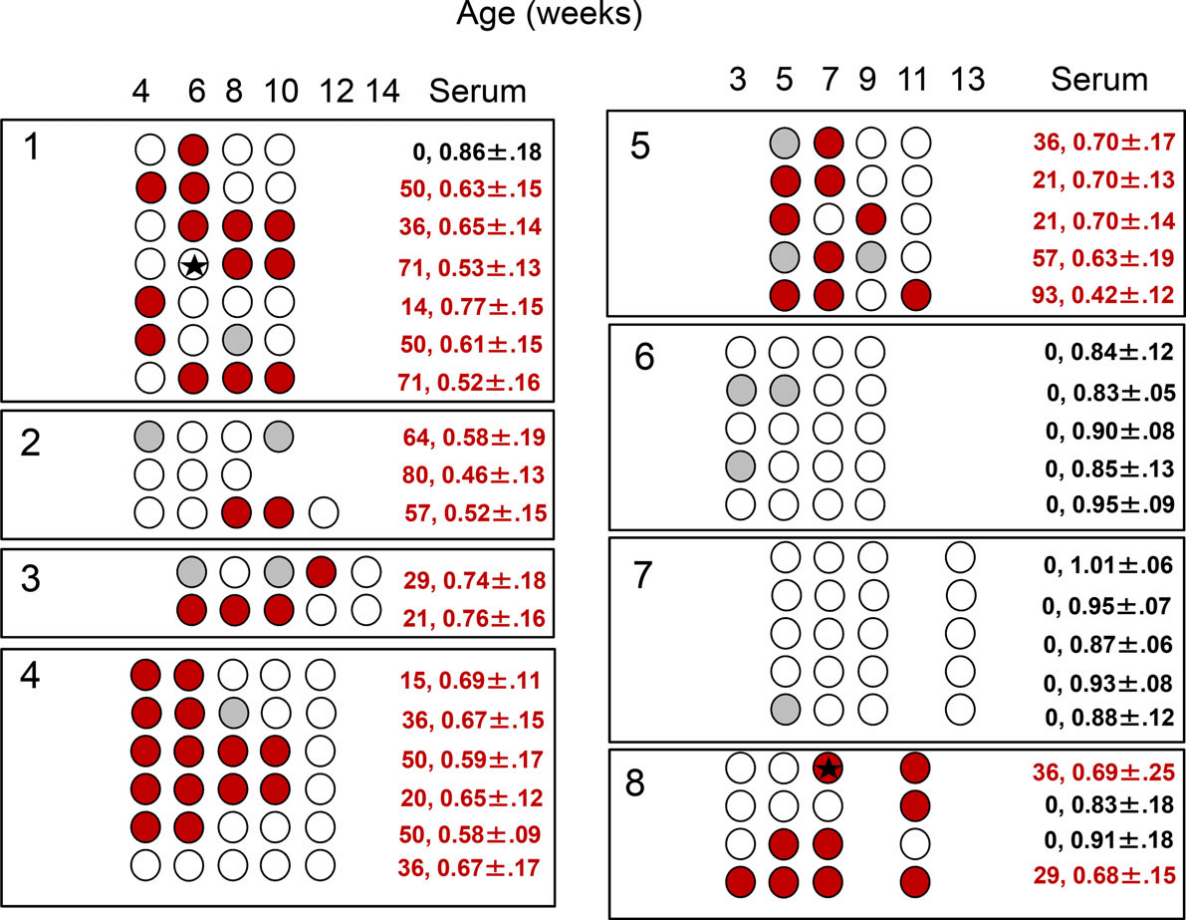
Anti‐Influenza A virus(IAV) antibody detection as determined by a blocking ELISA in pen‐based oral fluid (OF) samples in eight commercial pig farms sampled every 2 weeks for a maximum of five collection points. Sampled pens are indicated by circles. Empty circles represent negative samples. Positive samples are represented in red (S/N ratio ≤ 0.60) or grey (0.60 >  S/N ratio ≤ 0.65). Black stars represent IAV M gene RNA detection in OF samples. The numbers in the column designated ‘serum’ indicate the percentage of positive serum samples during the latest sample collection point and the S/N ratio ± standard deviation.

**Figure 2:**
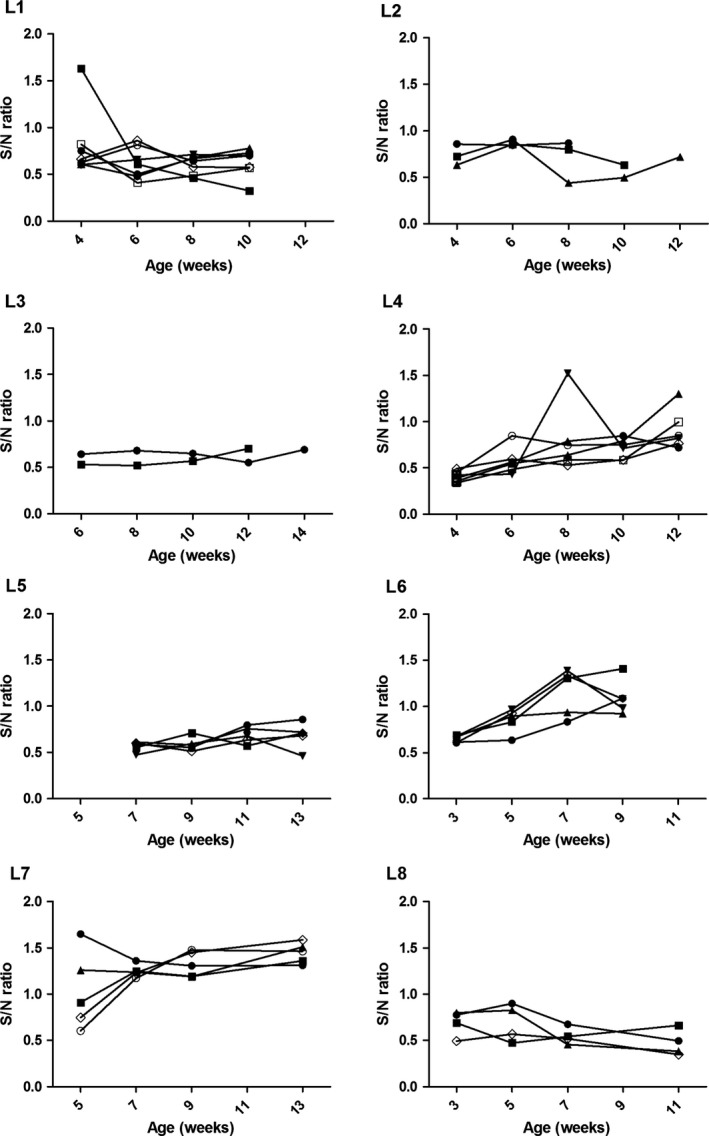
Anti‐ Influenza A virus(IAV) antibody sample to negative (S/N) ratios determined by a blocking ELISA on pen‐based oral fluid samples in eight commercial pig herds sampled every 2 weeks for a maximum of five collection points. An S/N ratio below 0.60 was considered positive.

Overall, 6/8 longitudinal herds were IAV antibody positive on OF and SER samples and IAV RNA was identified in 2/92 samples (2.2%) from two of the six positive herds, L1 and L8 (Fig. 1). Antibody kinetics in OF samples suggested a depletion of maternal antibodies from as early as 3–4 weeks (L6, L7) to 10–14 weeks of age (L3, L4) (Fig. 2). A rise in antibodies levels appeared to have occurred mostly around 8 weeks of age (L1, L2, L8). In herds in which IAV antibodies were detected in OF over time (L3, L4, L5, L8), both OF and SER classified a farm as positive, although at the last collection point a numerically higher number of pens were classified as positive based on SER (*n *=* *15) compared to OF (*n *=* *8). SER and OF samples from Herds L1 and L2 could not be compared due to a 2 week interval between the collections.

Herds L6 and L7 remained IAV antibodies negative for the duration of the study and both OF and SER samples agreed 100% on the pen‐based IAV classification (Fig. 1).

### IAV status in cross‐sectional herds

In cross‐sectional herds 17/19 herds were IAV antibody positive and 13 of the 17 positive herds had detectable IAV antibody levels in OF and SER samples. IAV antibodies were not detected in OF in four herds (C4, C10, C16 and C19) in which SER samples were positive. Two to five pens were tested in each of these herds and at least two SER samples were positive in 33.3–60% of the tested pens. The average OF S/N ratios ± standard deviation for 3–9, 10–14 and 18–20‐week‐old pigs were 0.82 ± 0.25, 0.81 ± 0.23, 0.65 ± 0.25, respectively. The decline in the average S/N ratios in both SER and OF samples indicated an active seroconversion in the 18–20‐week‐old pigs. All samples obtained from the positive herds were IAV RNA negative.

Two of the 19 cross‐sectional herds were IAV antibody negative based on OF and SER samples and IAV RNA was not detected in these two herds.

### Probability of detecting IAV antibodies using SER or OF samples

Using the cumulative data from the cross‐sectional and longitudinal studies, pig age was found to have a significant influence on the probability of detecting IAV antibody positive samples within a pen (*P *=* *0.04) with significant interaction between age and test type (*P *=* *0.02). Sample size had no effect on the probability of classifying a pen IAV positive under the study conditions. The probability rates for obtaining IAV antibody positive SER samples in different age groups are summarized in Table 2. When the results on 5–14 SER samples and one OF sample were compared for a pen, SER samples were more likely to be positive for IAV antibodies (Table 2). While there was a higher probability of obtaining a positive SER result compared to a positive OF result, this was at different significance levels for 3–9‐week‐old pigs (*P *=* *0.04), 10–14‐week‐old pigs (*P *<* *0.0001) and 18–20‐week‐old pigs (*P *=* *0.05). Percentage of sampling rates, number of animals per pen, pen size, number of pens sampled per herd, herd size or housing type did not influence these results. The probability of detecting a positive pen in the longitudinal study was 90% based on repeated OF sample testing over time (95% CI 0.58–0.98) and 85% based on a single SER sample collection (95% CI 0.42–0.98).

**Table 2:** Probabilities for detecting IAV antibodies with a nucleocapsid protein (NP)‐blocking ELISA in oral fluid (OF) or serum (SER) samples within a pen of pigs for different age groups with a 95% confidence interval

	3–9 weeks	10–14 weeks	18–20 weeks
Probability of a positive pen result based on a single OF sample	0.40 (0.23–0.60)^A^*	0.19 (0.08–0.40)^A^	0.67 (0.41–0.85)^A^
Probability of a positive pen result based on 5–14 SER samples	0.61 (0.37–0.80)^B^	0.93 (0.71–0.99)^B^	0.81 (0.63–0.91)^B^
Probability of a positive SER result for an individual animal	0.18 (0.09–0.33)	0.32 (0.17–0.51)	0.40 (0.26–0.57)

Different superscripts ^(A,B)^ within a column indicate a significant (*P* ≤ 0.05) different probability for detecting IAV antibodies in OF versus SER samples.

## Discussion

In this study anti‐IAV antibodies were detected in 88% (24/27) of the investigated herds, and in 35% (85/244) of the pens. In positive herds, 30–100% of the sampled pens were positive by OF or SER samples. The presence of IAV RNA in OF samples was confirmed in 8.3% (2/24) of the farms. When IAV antibody detection at the herd‐level was considered, results on SER and OF samples agreed for 23 of the 27 herds tested. Disagreement on herd classification, negative on OF samples and positive on SER samples, occurred in 4/27 herds. In these herds only 2–5 pens were sampled and a maximum of 1/6 to 3/6 SER samples were antibody positive within a pen.

Under the study conditions, the percentage of pigs sampled per pen offered a poor IAV status prediction. While the herds in this study were classified based on presence or absence of a detectable humoral immune response to IAV, the true rate of IAV positive animals within a pen or herd was unknown. Therefore, prevalence estimates could not be incorporated in the data modelling and this may partially explain the overall poor prediction. Previously, when anti‐PRRSv antibody levels in vaccinated pigs were investigated, OF samples had a positive rate of 61% with at least 4% SER prevalence in the same pen (Panyasing *et al*. 2013).

In this study, the probability of classifying a pen as anti‐IAV antibodies positive was overall lower for OF samples compared to SER samples when testing 5–14 SER samples per pen, regardless of the pen size. Pig age affected the probability to classify a pen as IAV positive and to detect IAV antibodies in OF samples. The age effect on the probability of detecting positive pens by SER was more remarkable for pigs up to 14 weeks of age than for pigs older than 18 weeks of age. Previously a similar age effect was observed for detection of anti‐PRRSv antibodies in which the overall agreement between OF and SER samples was 94% in 18–20‐week‐old pigs versus 72% in pigs from 3 to 14 week of age (Dawson 2015). This age effect could be partially explained by age dependent differences in pig representation in a OF sample defined as number of pig in a pen interacting with the rope for at least 20 s (Dawson 2015). Although there are reports that up to 75.5% of pigs in a pen interact with a rope within a 30 min OF collection interval, the results are based on observation in pens containing up to 28 6–12‐week‐old pigs (Vincent *et al*. 2014). It is worth noting that the pens sampled in the presented study held 20–540 pigs, with an average of 171 pigs per pen. A lower pig representation in an OF sample has been reported for larger pig groups (Dawson 2015). In pens containing 150–200 10‐week‐old pigs housed on straw, interaction with the rope increased from 42% with only one cotton rope to 74% when one rope per 18–25 pigs was provided (Dawson 2015). In pens containing 80–100 24‐week‐old pigs, pig interaction with a single rope (45–54%) did not change when offering additional ropes (Dawson 2015). Based on these findings, recommendations based on small pen sizes may not be adequate when applied to larger pens and the appropriate number of OF samples for larger pens needs further investigations.

The housing system can also affect the sample representativeness. The percentage of grow‐finish pigs interacting with the rope in straw‐based systems was lower (69%) when compared to systems with fully slated floors (81%), and interaction with the rope did not increase when up to four ropes were offered to pigs in pens with up to 24 animals (Seddon *et al*. 2012). In this study, no significant influence of percentage of sampling rates/pens/animals, pen size, herd size or housing type was identified. This may be partly due to the unstructured experimental design. The lack of effect of housing type on the agreement of antibody detection in OF compared to SER samples has been shown for PRRSv (Dawson 2015).

The assessment of IAV antibody responses over time in commercial swine populations could provide a cost‐effective data source on IAV infection status and herd immunity. Vaccination against IAV is not a common practice in European pigs and, when applied, usually only breeding animals are immunized (Kyriakis *et al*. 2011). The IAV antibody profile in nursery farms could also provide indirect information about the IAV status in breeding herds by the assessment of the presence of maternal antibody in weaned pigs. Monitoring maternal antibody decay in piglets could potentially aid in selection of the most appropriate vaccination time. In this study, the kinetics of antibodies in OF collected over time indicated a decay of maternal antibodies from 3–4 to 10 weeks of age. A rise of antibody levels, if detected, occurred at approximately 11–12 weeks of age. These results are in accordance with previous studies in other European countries using the HI assay (Simon‐Grife *et al*. 2012; Kyriakis *et al*. 2013; Rose *et al*. 2013). Although in this study SER samples gave an overall higher probability of identifying IAV antibody positive pens when 5–14 animals were sampled, the probability of detecting a positive pen was similar to OF samples when these were collected every 2 weeks as in the longitudinal study. This suggests that a more frequent regimen for OF sample collection should be used for IAV monitoring. The usage of a combination of a broadly reactive RT‐PCR and a serological assay as in this study may be particularly useful for screening purposes in populations with no apparent respiratory signs when the expected IAV prevalence may be low. Positive samples could then be further characterized using multiple specific serological and molecular assays to determine the circulating IAV subtype (Corzo *et al*. 2013).

Although this study was not designed to assess the occurrence of false positive results in OF samples, all but one pen classified as positive when using OF samples were also positive when using SER samples. It is worth noting that currently there is no commercially available ELISA for detection of IAV antibodies in OF samples. The protocol for the commercial NP ELISA used in this study was modified to be suitable for OF samples (Panyasing *et al*. 2016). An assay specifically optimized by the manufacturer for detection of PRRSv antibodies in OF samples has been shown to have a better diagnostic performance when compared to an *in‐house* modified protocol (IDEXX, 2012). In addition, changing the existing commercial NP blocking ELISA to an indirect ELISA format, by replacing the provided conjugate with anti‐porcine immunoglobulins, could perhaps improve its diagnostic performance (Olsen *et al*. 2013).

Results from this study are representative of the diagnostic challenges under field settings where the number of individual pigs sampled in a given pen is limited, the number of pigs per pen and per rope differs, and where the true prevalence of IAV antibodies within a pen is unknown. Under the study conditions, the detection rates of IAV antibodies were higher for SER samples when compared to OF samples but a more frequent OF sample collection could be used to account for this. In general, the probability of detecting IAV antibody positive OF samples was higher in older pigs (18–20 weeks) versus younger pigs (3–14 weeks) when compared with the detection rates on SER samples.

## Source of funding

Funding was provided by the Biotechnology and Biological Sciences Research Council (BBSRC) Institute Strategic Programme Grant awarded to the Roslin Institute (BB/J004324/1; BBS/E/D/20241864).

## Conflicts of interest

The authors declare no conflict of interest.

## Contribution

Study design and sample collection: LD, BS, RB. Sample testing: PFG. Statistical analysis: HB. Manuscript draft: PFG, TO. Revision and manuscript approval: PFG, HB, LD, BS, RB.
